# ReLoki: A Light-Weight Relative Localization System Based on UWB Antenna Arrays

**DOI:** 10.3390/s24165407

**Published:** 2024-08-21

**Authors:** Joseph Prince Mathew, Cameron Nowzari

**Affiliations:** Department Electrical and Computer Engineering, George Mason University, Fairfax, VA 22030, USA; jprincem@gmu.edu

**Keywords:** relative localization, UWB, ad hoc mobile beacons

## Abstract

Ultra Wide-Band (UWB) sensing has gained popularity in relative localization applications. Many localization solutions rely on using Time of Flight (ToF) sensing based on a beacon–tag system, which requires four or more beacons in the environment for 3D localization. A lesser researched option is using Angle of Arrival (AoA) readings obtained from UWB antenna pairs to perform relative localization. In this paper, we present a UWB platform called ReLoki that can be used for ranging and AoA-based relative localization in 3D. To enable AoA, ReLoki utilizes the geometry of antenna arrays. In this paper, we present a system design for localization estimates using a Regular Tetrahedral Array (RTA), Regular Orthogonal Array (ROA), and Uniform Square Array (USA). The use of a multi-antenna array enables fully onboard infrastructure-free relative localization between participating ReLoki modules. We also present studies demonstrating sub-50cm localization errors in indoor experiments, achieving performance close to current ToF-based systems, while offering the advantage of not relying on static infrastructure.

## 1. Introduction

**Figure 1:**
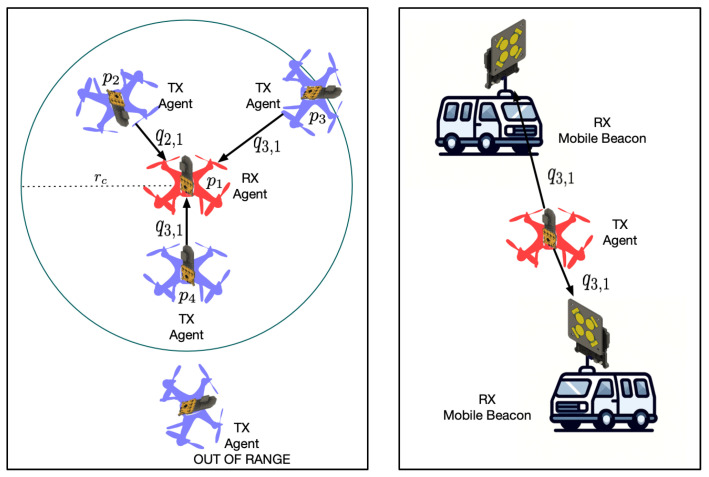
Illustration of the relative localization problem. On the left, we show ReLoki attached to an existing motion platform and capable of relative localization based on fully onboard sensing. Here, the RX agent senses the relative positions $q_{i,j}$ of the TX agents w.r.t its body frame whenever a message is received from *j*. On the right, we show the scenario where ReLoki can act as a mobile beacon. All beacons are capable of localizing a transmitting agent in 3D and adding more beacons will improve estimates.

Infrastructure-free ad hoc relative localization methods are needed in many applications that keep track of objects, robots, and even people. When neither a Global Positioning System (GPS), nor any infrastructure/landmarks are available for reliable global positioning, the agents must rely on relative localization to support missions like search-and-rescue or environmental monitoring, as shown in Figure 1. These systems can be further enhanced with ad hoc infrastructure setups, such as a localization beacon on a moving base station, which can be dynamically deployed in strategic locations. This paper proposes and validates a novel system using only Ultra-Wide Band (UWB) antenna arrays to relatively localize tags-to-tags or tags-to-mobile-beacons in a 3D environment.

We are focused on a light-weight, real-time distributed sensing solution for 3D relative localization that can seamlessly integrate (plug-and-play) into any existing multi-agent platform. Acquiring distance estimates based on Time of Flight (ToF) differences in UWB sensors is a commonly used method today [1,2,3,4]. A lesser explored area of interest for UWB systems is using multiple antenna arrays to leverage their Phase Difference of Arrival (PDoA) [5]. In this space, we propose ReLoki as a combined PDoA + ToF distributed relative localization system that is capable of estimating the transmitter’s location when two modules are communicating. For PDoA sensing, Reloki uses an on-board four-element antenna array. In this paper, we study the sensing performance of three specific antenna arrays: the Regular Orthogonal Array (ROA) and the Regular Tetrahedral Array (RTA) which are used for fully on-board 3D relative localization, and the Uniform Square array (USA) which is targeted for ad hoc beacon systems. In [6], the authors propose a theoretical methodology for localization with phase difference measurements [7,8,9,10]. We use a modified geometric approach for Angle of Arrival (AoA) estimation with the antenna arrays and show the performance characteristics in this paper.

### 1.1. Related Works

A common method of obtaining range measurements using UWB systems is the Tag–Anchor model [2,3,11]. Anchor nodes are set up at predefined locations and the Time Differences of Arrival (TDoAs) are used to estimate the relative position of these tags. Another implementation called Concurrent AoA estimation uses a single RX antenna as a tag and a multi-TX antenna array on the anchors situated at known locations in a room [12,13], with small delays between transmissions used for localization. These implementations are well suited for both indoor and outdoor deployments. By increasing the deployment density of tags and anchors, the localization accuracy can reach under 25cm. Additionally, since each tag only requires one UWB RF subsystem, the tags are light-weight and power-efficient, making them well suited to battery-operated systems. However, these systems need simultaneous observations from at least four beacons for full 3D localization. This limits the localization to areas specifically setup with anchors. Any ad hoc, infrastructure-free deployments are not possible with this architecture.

To remove the need for infrastructure, we can observe a multitude of sensor fusion methods [3,14,15,16,17], where the ranging measurements obtained from a single UWB TX/RX system are combined with some form of Simultaneous Localization and Mapping (SLAM) using an Inertial Measurement Unit (IMU) and/or visual data. This sensor fusion augments the UWB range-only measurements, enabling estimation of relative locations of neighbors without any ambiguity. The sensor fusion also allows for pose estimation, which provides full state information. While these systems generally have very good localization performance, they are far from plug-and-play and generally come with very complex computation requirements. Additionally, these systems require multiple readings from different points in the environment to estimate position, which contributes to an initial windup time before these sensors obtain lower errors in their positional estimates.

Multi-UWB range measurement systems have been developed to estimate the complete relative 2D positions (with pose) [18] and 3D positions [19,20,21] of robots. These systems use UWB antennas placed at pre-defined locations of the robot, i.e., the body of the robot is part of the antenna array structure. The difference in the ToF measurements on these different antennas corresponds to the AOA of the source. These systems work without any infrastructure and require only on-board processing for relative localization. They can additionally provide pose information with some assumptions. In these systems, the errors reduce with increasing separation between antennas. Hence, one of the main disadvantages is the large and bulky sub-frames needed to support the large separation between the antennas. For small sizes, the bearing estimation based on these ToF measurements has much higher errors than corresponding PDoA-based systems, as shown in [5]. Additionally, the 3D localization comes with ambiguity of translational position in one axis, as these systems use planar antenna arrays.

For full 3D relative localization, we can fuse ranging and bearing information to obtain a combined position estimate. PDoA estimation is an active area of research. The idea is to use phase differences measured by antennas separated by a distance of roughly half the carrier wavelength to estimate the AoA of the signal. Many of the works using narrow-band radios [22,23] consider planar antenna arrays with many antenna pairs to obtain very low bearing angle errors. However, these arrays comes with ambiguity in localization in at least one bearing estimate. Narrow-band RF localization also sufferers from multi-path effects leading to reduced localization performance in environments with many reflecting surfaces. Additionally, one of our requirements is a low-weight hardware system that can be integrated onto existing robotic platforms. Current UWB localization systems perform very accurate phase detection for their high-precision range estimation and they come in a very small package. Hence, in the case of our proposed ReLoki systems, a UWB-based PDoA-bearing angle estimation is coupled with a ToF range measurement. Looking at other UWB-based PDoA estimation methods, an azimuth-only AoA version is considered in [5], using an antenna array consisting of two antenna elements. However, this system exhibits azimuth ambiguity, meaning there is a one-to-two mapping of the measured phase difference in the range [−π,π] to the actual azimuth angle in the same range. This results in two possible mirrored azimuth estimates for a single phase difference value. The system in [24] is most similar to ours and is an extension of that in [5], which uses one more orthogonal pair (total of three antennas) to estimate both azimuth and elevation angle, but the same issue of azimuth ambiguity still exists. A major distinguishing factor of our work is exploring 3D antenna arrays for PDoA in UWB systems to eliminate this ambiguity observed in planar antenna arrays. Our previous work explored full 3D relative localization without azimuth ambiguity, showing the preliminary results for the RTA antenna [25]. In this work, we extend our preliminary results with improved experimental validation of the RTA antenna, present algorithms and validation for mobile ad hoc beacons using the USA antenna, and introduce a novel antenna design for ReLoki’s antenna array. When dealing with the antenna separation for the PDoA measurements, existing work on wrapped PDoA measurements for antennas with spacing greater than half the carrier wavelength [6,26] has shown theoretical performance gain over simple PDoA-based estimation without wrapping (for antenna spacing less than half the carrier wavelength), but this comes at the cost of using a computationally expensive non-convex search strategy to address phase ambiguity resolution. As a first proof of concept, we instead implement a simpler approach with antenna spacing less than half the carrier wavelength to facilitate the low weight of the hardware and faster estimation.

### 1.2. Contributions

The main contributions of the paper are as follows.

- First implementation of full 3D relative bearing estimation without ambiguity using 3D UWB antenna arrays. The current state of the art for UWB PDoA estimation using planar antenna arrays [5,24] performs bearing estimation with azimuth ambiguity.
- Hardware design of UWB module that supports interface with any four-element UWB antenna array that enables full 3D localization. Design details in GitHub https://github.com/denjos007/ReLoki-pub (accessed on 20 August 2024).

Other technical contributions include the following:

- First proof-of-concept 3D AoA estimation in light-weight hardware (weight: 65 g; dimensions: 20cm×9cm×3cm).
- Design and validation of UWB antenna for ReLoki system.
- Analysis of the localization performance of RTA, ROA, and USA antennas.
- Full-scale proof-of-concept experiments for a fully onboard relative sensing solution with two moving and one static agent, achieving an error tolerance of less than 25 cm for range estimates up to a range of 20m and $15^{\circ }$ for bearing estimates in the operational range of $\left(-60^{\circ },60^{\circ }\right)$ elevation.
- Full-scale proof-of-concept experiments for ad hoc beacon–tag system with two beacons and one tag showing a maximum localization error of 45cm in an indoor room of 10m×7m.

## 2. Multi-Agent Relative Localization via UWB

Consider a UWB-based transceiver pair (i,j), with *i* transmitting and *j* receiving data, and each transceiver consisting of four antennas. The 3D position of these transceivers are defined as $p_{i}\in R^{3}$ and $p_{j}\in R^{3}$. Note that these global positions will never be available to the agents. The goal of this system is to use this UWB system to obtain an estimate ${\hat{q}}_{i,j}$ of the true relative position qi,j≜ROBjj(pi−pj)∈R3 of the transmitter with respect to the body frame of the receiver, where ROBjj denotes the rotation from global to the body frame of the transceiver performing the relative localization sensing.

**Problem** **1** (3D relative position estimation). *Using the UWB sensing system mentioned above, find a co-designed Two-Way Ranging (TWR) + Phase Difference of Arrival (PDoA) pinging scheme and an online estimation algorithm that allows the receiving UWB system j to sense the relative position $q_{i,j}$ of the transmitter i, whenever i initiates a ranging request. The combined algorithm and hardware should minimize the expected relative localization error*

(1) $$\begin{array}{c} J_{i}^{err}=E[||q_{i,j}-{\hat{q}}_{i,j}||]. \end{array}$$

## 3. ReLoki Proof of Concept

ReLoki is our envisioned solution to the relative localization problem, capable of determining the distance and direction of an incoming transmission. Each ReLoki node consists of two parts: (1) an antenna array with specific geometry that is used to transmit/receive (TX/RX) data, and (2) a base platform consisting of four UWB Integrated Circuits (ICs) capable of computing the phase of arrival and time of arrival of an incoming signal, connected to a processing subsystem that performs all the computations.

**Figure 2:**
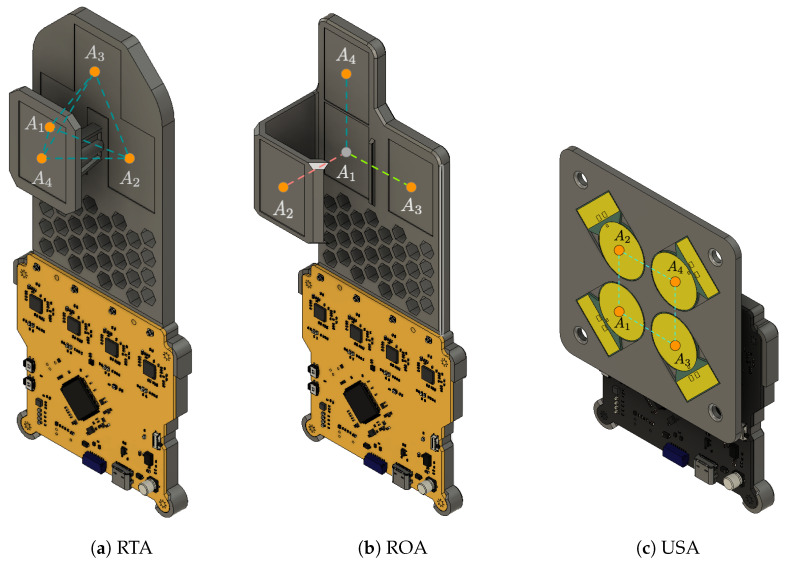
Illustration of the 4-antenna configurations that can be used with ReLoki. Here, we show the ROA, where the antennas are placed orthogonal w.r.t the central antenna, the RTA, where the antennas are placed at the vertices of a regular tetrahedron, and the USA, where the antennas are placed as a square on the same plane.

Full 3D localization requires at least four antennas, as seen in [6]. It should be noted that any four-element antenna array can be used with this system. In this paper, we will focus on the RTA, ROA, and USA arrays for their regular structures. An illustration of these antenna arrays is shown in Figure 2.

### 3.1. Relative Localization Using Four-Antenna Array

Localization of a source requires both ranging and direction/bearing information. Obtaining range measurements is straightforward. The ToF of a radio signal between two nodes is calculated using a well-established asynchronous Two-Way Ranging (TWR) protocol [27] between the nodes. This is known to provide centimeter-level accuracy [28,29].

To determine the direction to the source, we take advantage of the geometry of the specific receiving antenna array and transform the Phase Difference of Arrivals (PDoA) between different antenna pairs to the bearing angles. This can be performed each time a sensor *i* initiates what we call a Relative Position Ping (RPP).

For PDoA measurements, it is ideal to set the distance between all antenna pairs to half the wavelength of the carrier $\lambda_{c}/2$ to prevent wrapping (phase difference estimated goes over π or −π, thereby wrapping the opposite sign). Additionally, the carrier frequency should be in sync between all UWB transceivers on the receiver. Assuming we have the phase of arrival $\Phi_{i,j}^{n},n\in \left\{1,2,3,4\right\}$ for all four antennas in array, we can compute the phase difference $\Delta \Phi_{i,j}^{n,o}$ for all 42=6 antenna pairs as

(2) $$\begin{array}{c} \Delta \Phi_{i,j}^{n,o}=\Phi_{i,j}^{n}-\Phi_{i,j}^{o}+{\tilde{\Phi }}_{j}^{n,o},\;n\neq o. \end{array}$$

Ideally, the phase difference calculated will be zero when the source is perpendicular to the antenna pair. Our experiments showed that this is not the case in the hardware and all antenna pairs exhibited a phase difference bias. We use a bias cancellation term ${\tilde{\Phi }}_{j}^{n,o}$ to compensate for this. Bias compensation can be determined through a calibration experiment for each antenna pair. In this experiment, we first measure the phase of arrival without any bias for all possible bearing angles of the transmitter relative to the receiving antenna array. These measurements are then compared with the true angles expected based on the antenna geometry. The bias compensation ${\tilde{\Phi }}_{j}^{n,o}$ is found by minimizing the least squared error between the measured and true angles.

**Figure 3:**
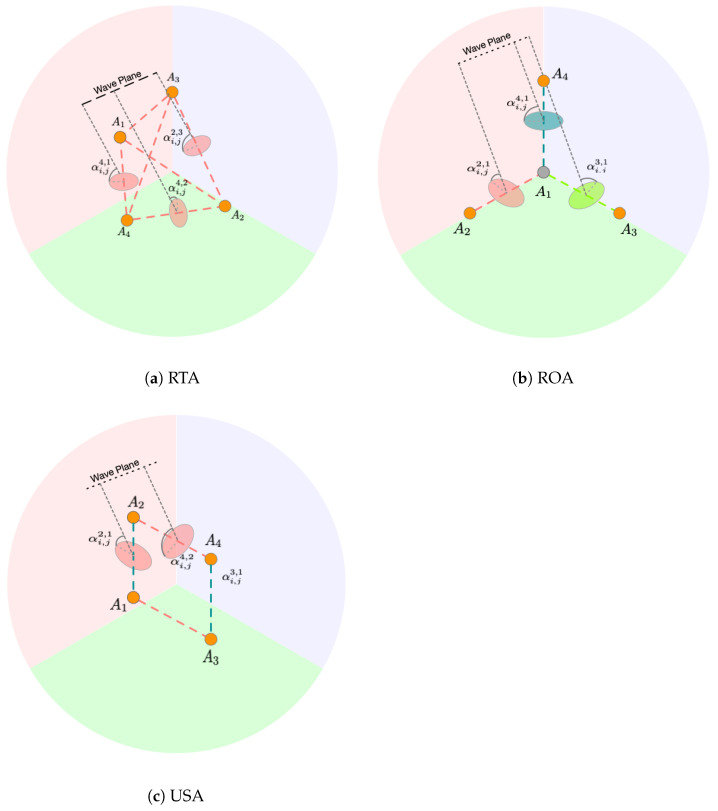
Illustration of angle of incidence for RTA, ROA, and USA Antennas. The angle of incidence measured is used for bearing estimates based on the specific geometry of the antenna array.

Subsequently, the angle of incidence $\alpha_{i,j}^{n,o}$ for all six antenna pairs is computed using (3). We show the illustration of angle of incidence for all three antenna arrays in Figure 3.

(3) $$\begin{array}{c} \alpha_{i,j}^{n,o}=\frac{1}{0.95}\arcsin\left(\frac{\Delta \Phi_{i,j}^{n,o}}{\pi }\right),\;n\neq o. \end{array}$$

Notably, a fraction of $\frac{1}{0.95}$ is used. In the actual design of the antenna array, the spacing between antennas is set to $0.95\lambda_{c}/2$. This spacing is chosen to ensure that the calculated phase differences remain within the [−π,π] range, mitigating errors caused by noise in the phase calculation algorithm, as mentioned in [5].

Using the six incidence angle $\alpha_{i,j}^{n,o}$ measurements and the specific geometry of the antenna array, we can obtain the unit vector ${\hat{\upsilon }}_{i,j}=\left[{\hat{\upsilon }}_{i,j}^{x},{\hat{\upsilon }}_{i,j}^{y},{\hat{\upsilon }}_{i,j}^{z}\right]$. Below, we show the specific geometric transformations for each antenna array.

#### 3.1.1. RTA Transformation

The RTA antenna has the antenna elements at the vertices of a regular tetrahedron. Since all six antenna pair separations are within half the carrier wavelength $\lambda_{c}/2$, there is no phase wrapping in any antenna pair. The transformation for the RTA array is

(4) $$\begin{array}{c} \left[\begin{array}{ccc} 1 & 0 & 0\\ \left(-1/2\right) & 0 & \left(\sqrt{3}/2\right)\\ \left(-1/2\right) & 0 & -(\sqrt{3}/2)\\ \left(1/2\right) & \left(\sqrt{3}/6\right) & \left(\sqrt{6}/3\right)\\ \left(1/2\right) & \left(\sqrt{3}/6\right) & -(\sqrt{6}/3)\\ 0 & \left(\sqrt{6}/3\right) & \left(1/\sqrt{3}\right) \end{array}\right]\left[\begin{array}{c} {\hat{\upsilon }}_{i,j}^{x-}\\ {\hat{\upsilon }}_{i,j}^{y-}\\ {\hat{\upsilon }}_{i,j}^{z-} \end{array}\right]=\left[\begin{array}{c} \sin(\alpha_{i,j}^{2,1})\\ \sin(\alpha_{i,j}^{3,2})\\ \sin(\alpha_{i,j}^{1,3})\\ \sin(\alpha_{i,j}^{4,1})\\ \sin(\alpha_{i,j}^{4,2})\\ \sin(\alpha_{i,j}^{4,3}) \end{array}\right]. \end{array}$$

where ${\hat{\upsilon }}_{i,j}^{x-}$, ${\hat{\upsilon }}_{i,j}^{y-}$, and ${\hat{\upsilon }}_{i,j}^{z-}$ are the noisy estimates of the *x*, *y*, and *z* components of the bearing vector, respectively.

In ideal, noise-free conditions, all six equations should converge to a unique solution. However, with noisy measurements, the system becomes over-constrained. For UWB modules, noise is directly correlated with the $|\alpha_{i,j}^{n,o}|$ [5], i.e., when the magnitude is low, the incident angle estimate has minimal error, but estimates above $70^{\circ }$ exhibit higher errors. Therefore, for a given set of phase differences, we exclude from (4) the row corresponding to the antenna pair with a very high phase difference $|\Delta \Phi_{i,j}^{n,o}|>\Delta {\bar{\Phi }}_{i,j}^{n,o}$. We determine that a threshold of  $\Delta {\bar{\Phi }}_{i,j}^{n,o}=165^{\circ }$ works well with experimentation. We then use the pseudo-inverse of the left-hand matrix, based on the remaining pairs with valid phase differences, to approximately solve (4) and obtain the noisy direction estimate.

#### 3.1.2. ROA Transformation

In the orthogonal array, the antennas are arranged so that pairs of antennas are along the cardinal axis. Subsequently, only the elements that are in line with the cardinal axis have an antenna separation of $\lambda_{c}/2$, making the phase difference estimated at these pairs proportional to actual angle of incidence. The other pairs will exhibit phase wrapping, and therefore are discarded. Hence, the transformation for ROA antenna is

(5) $$\begin{array}{c} \left[\begin{array}{ccc} 1 & 0 & 0\\ 0 & 1 & 0\\ 0 & 0 & 1 \end{array}\right]\left[\begin{array}{c} {\hat{\upsilon }}_{i,j}^{x-}\\ {\hat{\upsilon }}_{i,j}^{y-}\\ {\hat{\upsilon }}_{i,j}^{z-} \end{array}\right]=\left[\begin{array}{c} \sin(\alpha_{i,j}^{2,1})\\ \sin(\alpha_{i,j}^{3,1})\\ \sin(\alpha_{i,j}^{4,1}) \end{array}\right]. \end{array}$$

It is important to note that, in the case of the ROA array, the transformation simplifies to a direct assignment of the sine of the incident angle, as the antenna pairs are aligned with the cardinal axes.

#### 3.1.3. USA Transformation

In both the RTA and ROA arrays, the antenna pairs were arranged so that only three antennas were in the same plane, with at least one antenna positioned outside this plane. This arrangement enabled unambiguous 3D localization. In contrast, the square array has all antenna pairs lying on a single plane, resulting in a ± ambiguity along the axis perpendicular to this plane. In this particular setup, all antennas are on the YZ plane, causing a ± ambiguity for the X-axis coordinates. Consequently, we will limit the X-axis localization readings to only positive values.

With all four antennas in the same plane, the antenna array includes redundant pairs along the X- and Z-axes. This configuration allows us to average the readings from both pairs, providing a more accurate estimate of the angle of incidence and thereby reducing bearing errors. Hence, the transformation for USA array is

(6a) $$\begin{array}{cc} \; & \left[\begin{array}{ccc} 1 & 0 & 0\\ 0 & 1 & 0\\ 0 & 0 & 1 \end{array}\right]\left[\begin{array}{c} {\hat{\upsilon }}_{i,j}^{x-}\\ {\hat{\upsilon }}_{i,j}^{y-}\\ {\hat{\upsilon }}_{i,j}^{z-} \end{array}\right]=\left[\begin{array}{c} \sqrt{\sin^{2}\left(\alpha_{i,j}^{y}\right)+\sin^{2}\left(\alpha_{i,j}^{z}\right)}\\ \sin(\alpha_{i,j}^{y})\\ \sin(\alpha_{i,j}^{z}) \end{array}\right] \end{array}$$

(6b) $$\begin{array}{cc} \; & \alpha_{i,j}^{y}=\gamma_{\mathrm{azm}}\alpha_{i,j}^{1,3}+\left(1-\gamma_{\mathrm{azm}}\right)\alpha_{i,j}^{2,4} \end{array}$$

(6c) $$\begin{array}{cc} \; & \alpha_{i,j}^{y}=\gamma_{\mathrm{ele}}\alpha_{i,j}^{1,2}+\left(1-\gamma_{\mathrm{ele}}\right)\alpha_{i,j}^{3,4} \end{array}$$

(6d) $$\begin{array}{cc} \; & \gamma_{\mathrm{azm}}=\left\{\begin{array}{c} 0.9,\;\mathrm{if}\;\alpha_{i,j}^{1,3}<-60^{\circ }\\ 0.1,\;\mathrm{if}\;\alpha_{i,j}^{2,4}>60^{\circ }\\ 0.5,\;\mathrm{otherwise} \end{array}\right.,\;\gamma_{\mathrm{ele}}=\left\{\begin{array}{c} 0.9,\;\mathrm{if}\;\alpha_{i,j}^{1,2}<-60^{\circ }\\ 0.1,\;\mathrm{if}\;\alpha_{i,j}^{3,4}>60^{\circ }\\ 0.5,\;\mathrm{otherwise} \end{array}\right. \end{array}$$

**Figure 4:**
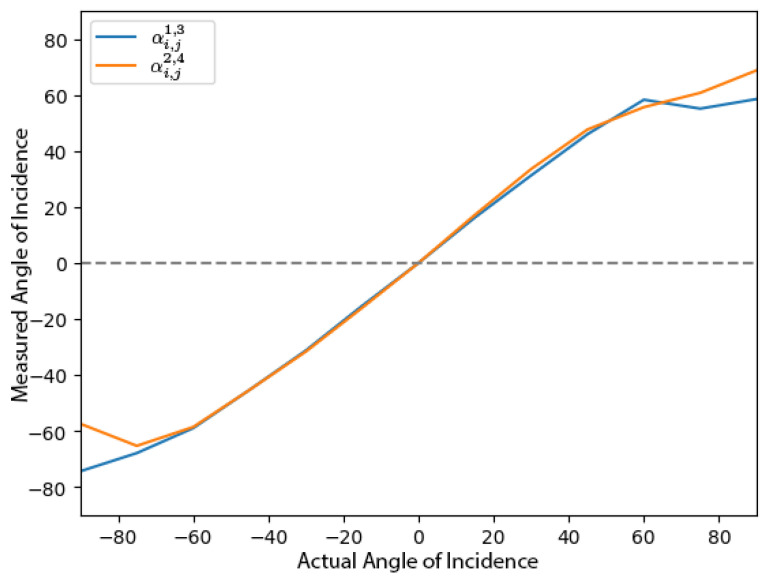
Angle of incidence measured for the redundant pairs. Here, the measured value is the average of 20 readings. The plot shows the saturation of the angle of incidence measured over $60^{\circ }$ in one pair and under $-60^{\circ }$ in the other.

In the USA antenna array, we use a weighted average to combine the angles of incidence from redundant antenna pairs. The weight for the azimuth pairs is defined by $\gamma_{\mathrm{azm}}$ and that for the elevation pairs is defined by $\gamma_{\mathrm{ele}}$. During experiments, we observed that for angles above $60^{\circ }$, one antenna pair exhibited saturation in the measured angle of incidence, while the other pair showed a complementary saturation for angles below $-60^{\circ }$. This effect is illustrated in Figure 4. The weighted filter assigns less weight to the saturated measurements, making the values at higher angle of incidences more accurate.

#### 3.1.4. Normalization

Due to the inherent noise in the UWB antenna pairs, the unit vectors computed may not have unit magnitude and hence we normalize the vector as

(7) $$\begin{array}{cc} {\hat{\upsilon }}_{i,j}^{m} & =\frac{{\hat{\upsilon }}_{i,j}^{m-}}{\sqrt{\sum_{s\in x,y,z}{\left({\hat{\upsilon }}_{i,j}^{s-}\right)}^{2}}}. \end{array}$$

The full AoA estimation relative localization process is formalized in Algorithm 1. With the range measurement ${\hat{r}}_{i,j}$ obtained from TWR and the direction ${\hat{\upsilon }}_{i,j}$ obtained from Algorithm 1, we obtain the estimated relative localization of the system as ${\hat{q}}_{i,j}=\left[{\hat{r}}_{i,j}{\hat{\upsilon }}_{i,j}^{x},{\hat{r}}_{i,j}{\hat{\upsilon }}_{i,j}^{y},{\hat{r}}_{i,j}{\hat{\upsilon }}_{i,j}^{z}\right]$.
**Algorithm 1** Angle-of-Arrival (AoA) Estimation.     **Input** phase of arrival at each antenna $\Phi_{i,j}^{n}$     **Output** unit vector bearing of neighbor ${\hat{\upsilon }}_{i,j}$1:Calculate the phase difference $\Delta \Phi_{i,j}^{n,o}$ with the bias compensation using (2).2:Calculate the incidence angle $\alpha_{i,j}^{n,o}$ at each antenna pair using (3).3:Obtain the raw estimate of the unit-bearing vector ${\hat{\upsilon }}_{i,j}^{-}$ using (4), (5), or (6) depending on the antenna array used.4:Normalize the unit vector ${\hat{\upsilon }}_{i,j}$ to length 1 using (7). **return** ${\hat{\upsilon }}_{i,j}$

### 3.2. Messaging Protocol

**Figure 5:**
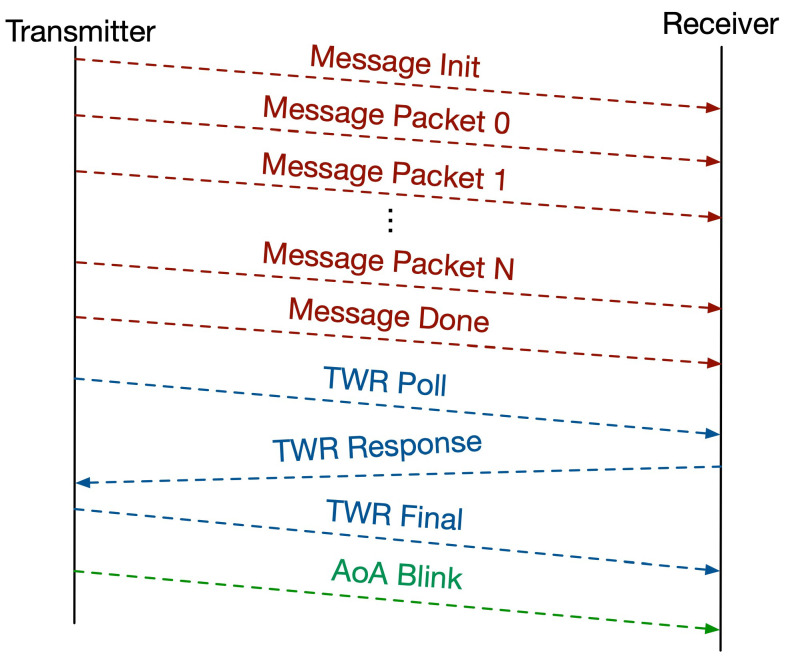
Timing diagram showing the different phases of transmissions. Message Transfer phase is shown in red, TWR Ranging phase in blue, and AoA Blink phase in green.

Here, we discuss the specific messaging protocol ReLoki uses for simultaneous communication and localization. Only one antenna $A_{1}$ is used when ReLoki acts as the transmitter. All transactions begin with a request to initialize an RPP called Message Init. Subsequent data transfer consists of three phases: Message Transfer, TWR Ranging, and AOA Blink, as shown in Figure 5. In the Message Transfer phase, the message to be transmitted is properly formatted to a 802.15.4A packet and sent to the receiver. In a typical 802.15.4A transmission, the maximum payload is 127 bytes of data. Hence, we split the original message to packets of size 120 bytes and transmit the data. The extra 7 bytes can be used for packet header data and Cyclic Redundancy Check (CRC). The second phase is the TWR Ranging phase, implemented using the TWR protocol mentioned in Section 3.1, and estimates the distance to the source ${\hat{r}}_{i,j}$. The receiver uses only its $A_{1}$ antenna for the Message Transfer and TWR Ranging phases mentioned above. The third phase is the AoA Blink phase, used to estimate the bearing angle of the transmitter from the receiver ${\hat{\upsilon }}_{i,j}^{m}$, and here all four antennas are used on the receiver side. The RPP is completed upon successful reception of the messages from all three phases at the receiver. The transaction is terminated when a failure is detected at any phase of the protocol.

### 3.3. ReLoki Hardware Platform

Our proof-of-concept ReLoki system consists of a set of four custom antennas interfaced with a custom controller board, which in turn consists of the UWB RF system and an onboard processor. We discuss the details of the hardware design in this section.

#### 3.3.1. Antenna Design

**Figure 6:**
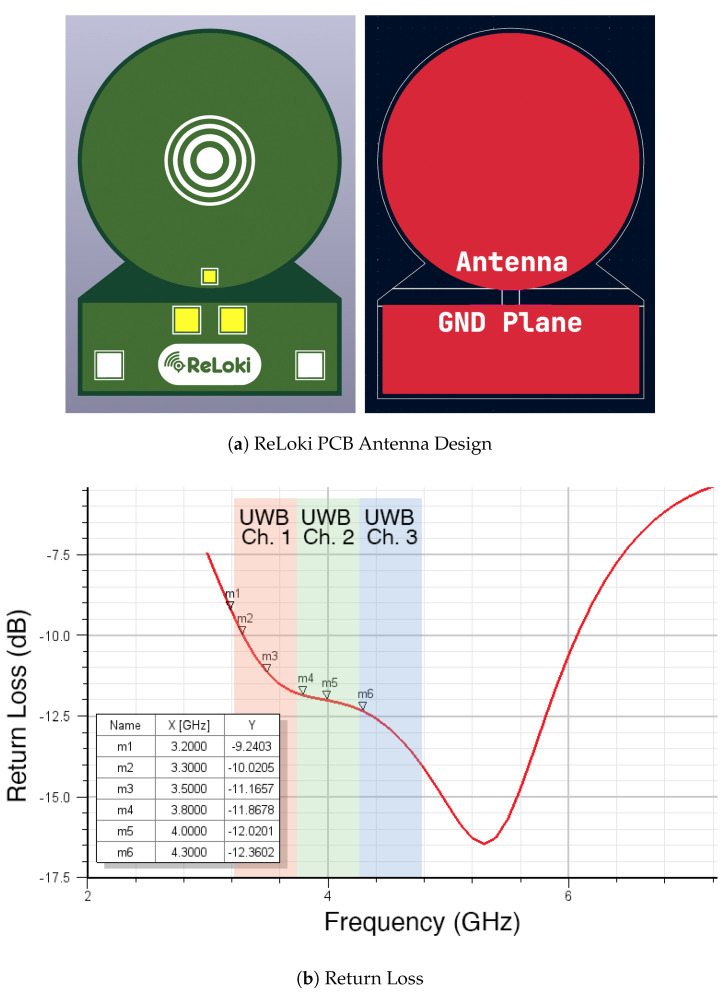

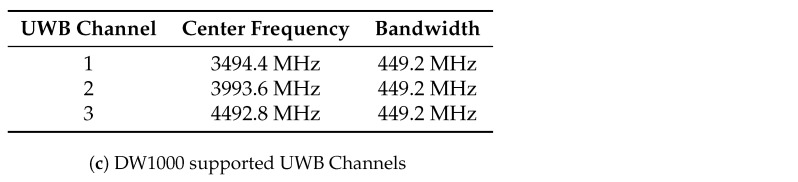
Single-antenna design for ReLoki. (**a**) Finished PCB antenna along with the copper plane showing the circular patch antenna and the ground plane. (**b**) Return loss for the designed antenna showing less than −10dB return loss in almost all the UWB band for Ch. 1, 2, and 3. (**c**) Center frequency and the bandwidth of the UWB bands supported by proposed antenna and DW1000.

All antennas interfacing with the control board in the ReLoki system have a PCB patch antenna with a circular geometry and an associated rectangular ground plane. The circular signal patch has a radius of 11mm and the rectangular patch’s dimension is 22mm×7.5mm. The separation between signal and ground patches is 1.5mm. The design of the antenna is shown in Figure 6. The antenna is designed for best performance in the UWB channels 1, 2, and 3, as evidenced by the return loss in Figure 6b. We chose the channels with the lowest center frequency to obtain the highest possible separation between antennas in the array. This allows for higher tolerance to manufacturing errors. Hence, all testing for our system was conducted with channel 1.

The individual antennas are subsequently attached to a 3D-printed base structure to create the specific four-antenna array structure. This manufacturing technique keeps the weight of the antenna to a minimum with very little interference to the received signal. Any antenna array can be manufactured with a combination of the PCB patch antenna and the 3D-printed substructure.

#### 3.3.2. Controller Design

**Figure 7:**
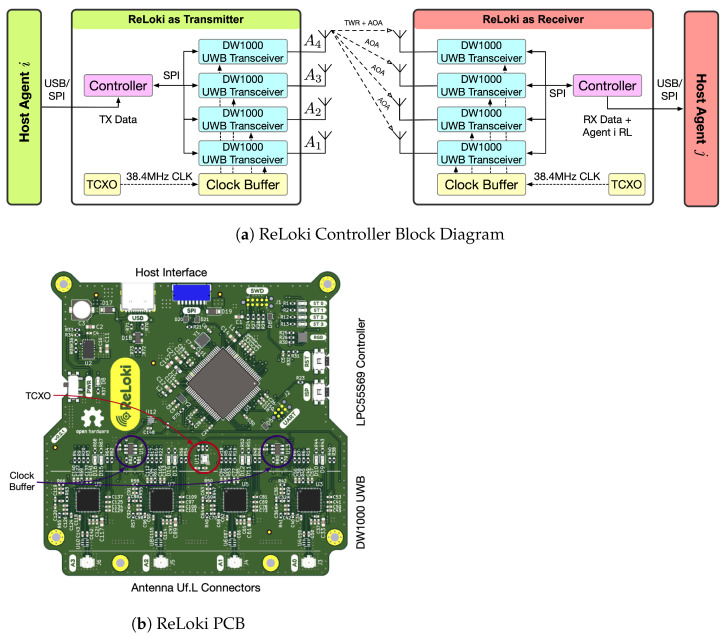
ReLoki controller design. (**a**) ReLoki hardware block diagram showing the components. Here, we start with the host *i* initiating a communication request. ReLoki connects to host *i* and transmits the data. The information is transferred to the receiving ReLoki where it is then combined with the estimated localization data. Finally, the data are sent to the receiving host *j*. (**b**) ReLoki PCB design showcasing the different components mentioned in the block diagram.

Each antenna in the array needs to interface with an RF subsystem to estimate the ToF and the phase of arrival. DW1000 is one of the most popular UWB RF Integrated Circuits (ICs) capable of both ToF and PDoA estimation. Each ReLoki controller uses four DW1000 ICs, all interfaced with an LPC55S69 microcontroller for data processing. When computing the PDoA between antenna pairs, the error in phase difference estimates increases with increasing skew of the carrier wave generated by each DW1000 IC. We use a single external clock source, the ATX-13-38400 at 38.4 MHz, to ensure that the carriers generated by all four DW1000 ICs are in sync. The clock is distributed to all DW1000 ICs through a clock buffer, the PL133. Additionally, all clock traces on the PCB are length matched to 0.1mm. These design parameters translate to a maximum clock skew of 34ps. Both the distribution buffer and the length matching allows for a low clock skew between DW1000 ICs. To allow the connection of any four-element antenna array, the controller utilizes Uf.L connectors. All computations for setting up data transfer and performing relative localization estimation are implemented on this controller platform. We show a block diagram of the system and the PCB in Figure 7. The total weight of one ReLoki module is only 65g including PCB, antennas, and the 3D-printed antenna holders.

When all four antennas on the controller receive data from a single source, the DW1000 ICs will compute the time series of the Complex Impulse Response (CIR) of the received signal and store it in the in the accumulator memory [5]. The phase of arrival $\Phi_{i,j}^{n}$ of the first path is calculated as

(8) $$\begin{array}{c} \Phi_{i,j}^{n}=\arctan\left(I_{n}\left(t_{i,j}^{n.\mathrm{fp}}\right)+i.Q_{n}\left(t_{i,j}^{n.\mathrm{fp}}\right)\right)-\beta_{j}^{n}, \end{array}$$

where $t_{i,j}^{n.\mathrm{fp}}$ denotes the time in the accumulated memory of the DW1000 which it has determined to be the first path of the received signal. $I_{n}\left(.\right)$ and $Q_{n}\left(.\right)$ denote the real and complex magnitudes of the received signal in the accumulated memory. Additionally, a correction factor, called the Synchronous Frame Detection (SFD) angle $\beta_{j}^{n}$, is applied to the calculated phase. This phase correction is an artifact of the first path detection in the DW1000 module [5].

When a host connected to ReLoki *i* wants to send a message to its neighbors, it sends the message to the ReLoki through its host interface. The transmitter ReLoki initiates the RPP transaction if the airway is clear. Upon completion of the RPP, the receiving ReLoki will have both the received message and the estimated relative position of the transmitter. It then combines both into a single message for the host and notifies the connected host of the received message. The host can then read these data and use them as needed, making ReLoki a plug-and-play localization solution for any existing system.

**Remark** **1.** ***Inter-module clock synchronization:*** *In some of the TDoA-based UWB positioning solutions, complex timing synchronization between beacons is mandated to properly compute the time difference. In the ReLoki system, the TWR ranging protocol does not require any timing synchronization between modules. Additionally, the phase difference is calculated between antenna pairs on the same module. Consequently, we only need clock synchronization between DW1000 ICs on the same module. Thus, for both TWR and AoA estimations, ReLoki does not mandate clock synchronization between two ReLoki modules.*

### 3.4. Muti-Agent and Multi-Beacon Support

Only one sender–receiver pair can perform the UWB-based position sensing at any given time. To localize multiple modules, a form of time multiplexing must be implemented. The default state of all ReLoki modules is to wait for Message Init. Any valid 802.15.4 packet will trigger the receive state of the UWB module. This can be used as a way for all modules to gauge the air traffic within their communication range. Thus, for any ReLoki wanting to transmit a message, the transmission will be delayed for a random time if that ReLoki is already participating as a receiving agent. The transmission happens only if the airway is clear at the end of this timeout. This is a specific implementation of carrier sensing based on the UWB states that is supported by DW1000, which we leverage for Carrier Sense Multiple Access (CSMA). Additionally, specific ID-based filtering is implemented in the handshake message so that the data can be sent to a specific receiver. The implementation of both CSMA and ID-based filtering ensures that there is only one RX-TX pair active at any given time.

## 4. Performance Analysis and Experiments

We test the performance of the ReLoki on two fronts. First, we experimentally measure the errors in estimation and generate a covariance map. Second, to demonstrate the real-world performance of the system, we set up experiments where the ReLoki system is mounted on physical robots and remotely controlled.

### 4.1. Covariance Maps

**Figure 8:**
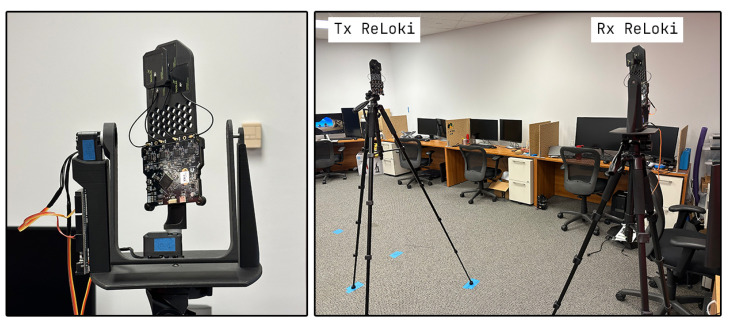
ReLoki experimental setup for covariance measurement. On the left, we have the pan and tilt mechanism and on the right we have the test setup for the 1.5m range from source.

The covariance map for ReLoki is the map of the expected error at different regions of the sensing domain. Having a lower value in this covariance map will translate to a lower localization error. To determine the covariance map, we set up an experiment where one ReLoki is mounted on a pan–tilt setup, as shown in Figure 8, which allows us to test all possible orientations the transmitter will have with respect to the receiver. We take 50 readings for multiple pan–tilt range combinations. The pan range for the RTA and ROA antennas is $\left[-180^{\circ },180^{\circ }\right]$ and for the USA antenna it is $\left[-90^{\circ },90^{\circ }\right]$ due to the X-axis ambiguity mentioned in Section 3.1. The elevation ranges for all antenna arrays are in the range $\left[-90^{\circ },90^{\circ }\right]$ in steps of $15^{\circ }$ and ranges from 1.5m to 7.5m in steps of 1m.

The covariance map is obtained using two factors. First, we obtain the average error from the ground truth data ${\mathrm{Cov}}^{e}\left(q_{i,j}^{m}\right):=E{\left[{\hat{q}}_{i,j}^{m}-q_{i,j}^{m}\right]}^{2}$ for all 50 readings taken at the relative range of pan–tilt $q_{i,j}^{m}$. We obtain the bearing ground truth from the pan and tilt mechanism, which moves the ReLoki system to a specific relative bearing with respect to the source. The range ground truth is obtained from manually measuring the distance to this source in the experimental run. We also obtain the covariance (spread) of these 50 readings ${\mathrm{Cov}}^{\sigma }\left(q_{i,j}^{m}\right)$ at the set relative position. The final error measured is a very conservative estimate of the actual covariance and computed as

(9) $$\begin{array}{c} \mathrm{Cov}\left(q_{i,j}^{m}\right)=\det\left|\mathrm{diag}\left({\mathrm{Cov}}^{e}\left(q_{i,j}^{m}\right)\right)+{\mathrm{Cov}}^{\sigma }\left(q_{i,j}^{m}\right)\right|. \end{array}$$

A lower value here means a lower cost in (1).

#### 4.1.1. RTA Antenna

**Figure 9:**
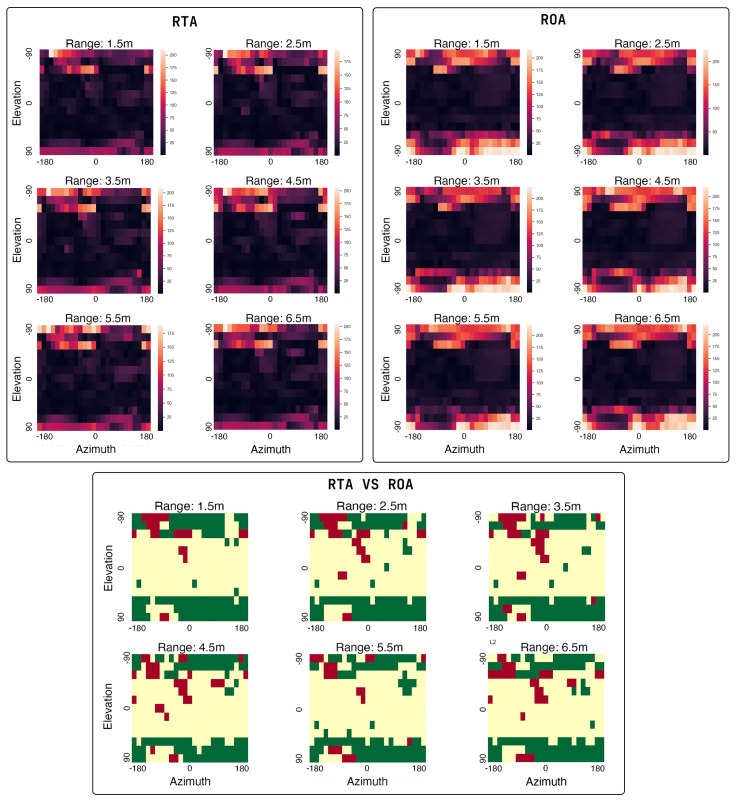
Covariance maps for RTA and ROA antennas. In the top left we have RTA and in the top right we have the ROA array. A darker color means lower error. On the bottom, we show the comparison of RTA antenna array to the ROA antenna array. Here, green boxes represent lower errors for RTA and red represents lower errors for ROA. Yellow a represents comparable performance (combined azimuth and elevation difference within $10^{\circ }$) between both.

We show the measured covariance map for the RTA antenna in Figure 9. Looking at the covariance maps, we can see that there are very high errors of more than $100^{\circ }$ at elevation angles $-90^{\circ }$, $-75^{\circ }$ and $-60^{\circ }$. These errors are attributed to the ReLoki controller electronics being in the path of the incoming signal at these angles, causing skewed estimates. We also see higher errors at an elevation angle of $90^{\circ }$, with an error of up to $80^{\circ }$, which may be due to the antenna characteristics of the chosen antenna providing very little illumination from the source at this relative angle. Additionally, we see that at elevation angles $-15^{\circ }$–$-45^{\circ }$ and close to an azimuth of −30 and +60 there are higher errors in the range of up to $45^{\circ }$ deviation from actual values. We attribute this to the ground plane of the protruding antenna $A_{4}$ interfering with phase calculation in antenna $A_{1}$ and $A_{2}$. In other regions, the error in azimuth and elevation angles are within $15^{\circ }$ and will provide good localization performance. The range measurements during the TWR ranging phase come to an accuracy of around 25cm on average up to a maximum range of 20m.

#### 4.1.2. ROA Antenna

We also show the covariance maps of the ROA array in Figure 9. Similar to the errors observed in the RTA array, we notice high errors at elevation angles below $-60^{\circ }$ due to obstructions caused by the electronics. Likewise, we observe similarly high errors at angles above $60^{\circ }$. This may be attributed to very poor RF illumination of the receiving antenna array from the source. Outside the previously mentioned elevation angles, we observe excellent performance for bearing localization, with errors within $15^{\circ }$. We also see a very similar performance in ranging measurements between the ROA and RTA arrays as both use the same setup and algorithm for ranging.

We additionally show the comparison of the RTA array to the ROA array in Figure 9. Here, we can observe a significant reduction in errors at higher elevation angles for the RTA antenna as noted by the green regions in the figure. At lower elevation angles, the errors are low in both implementations, with very small differences except for a few conditions, as mentioned above. We can conclude that in applications where the elevation angles between agents are restricted to lower values, the orthogonal array provides a slightly better estimate in the operational regions of such application. However, for a full 3D localization system, the RTA array provides better overall performance than the orthogonal array over the entire operational region.

#### 4.1.3. USA Antenna

**Figure 10:**
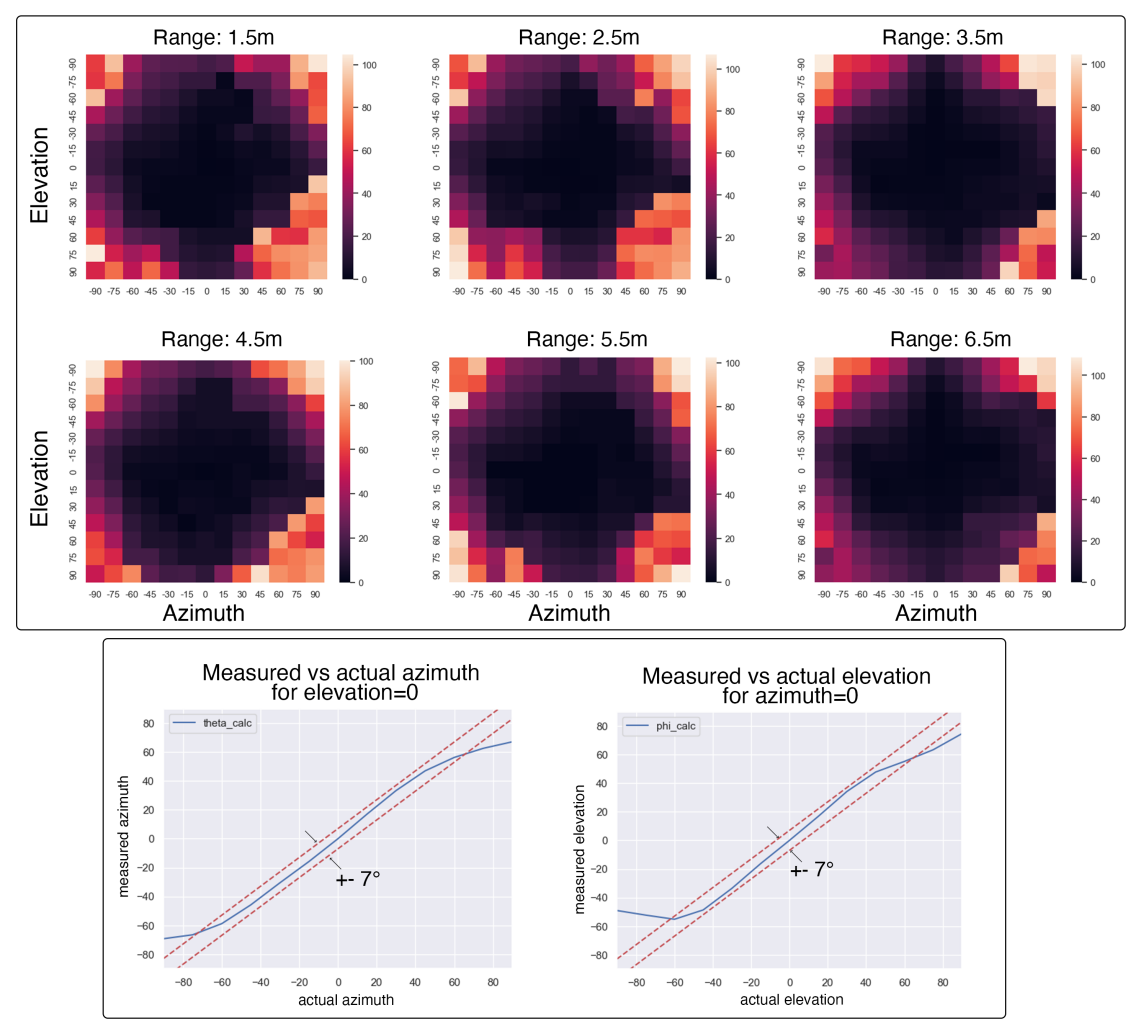
Covariance map for USA antenna. On the top, we have the covariance maps, with darker colors showing lower errors in localization and lighter colors showing higher errors in localization. On the bottom, we show the average of measured vs actual values for azimuth and elevation for 50 readings at a given pan–tilt pair.

We show the covariance maps for the USA antenna array in Figure 10. In this antenna array, we notice an overall reduction in error as compared to the RTA and ROA antenna arrays. The redundant antenna pair, in the USA antenna, provides significant reduction in localization errors. When the azimuth and elevation angles are between $-45^{\circ }$ and $45^{\circ }$, the errors in localization are within $10^{\circ }$. With bearing angles approaching higher values, the errors go as high as over $100^{\circ }$. This is consistent with the overall AOA performance of individual antenna pairs. Hence, the USA antenna is more suited for beacon–tag applications with the advantage that a single beacon can fully localize a tag in its operational domain. The range measurements and the errors were very similar to the ROA and RTA antenna pairs.

### 4.2. Maximum Operating Frequency

We have observed that each RPP between agents take up to 46ms per transmission based on the clock output observed between transfers. This timing includes handshake, one data packet, TWR, and finally, AOA transactions, which are the smallest transactions possible. Hence, we can estimate at most 20 transactions between agents per second, which decreases with the size of data being transmitted. So, for $N_{s}$ sensors the max frequency for each sensor will be $20/(N_{s}-1)$.

### 4.3. Robot Localization

To show the expected real-world performance, we conduct localization experiments with the ReLoki system in experimental scenarios. In the first scenario, the ReLoki is mounted on Turtle bots. These experiments showcase the infrastructure-free 3D localization. In the second scenario, the ReLoki module is handheld by a human operator, which demonstrates the use of ReLoki as a mobile beacon. The system performs the associated relative localization task and the localization performance is compared to the ground truth data.

#### 4.3.1. RTA Antenna

**Figure 11:**
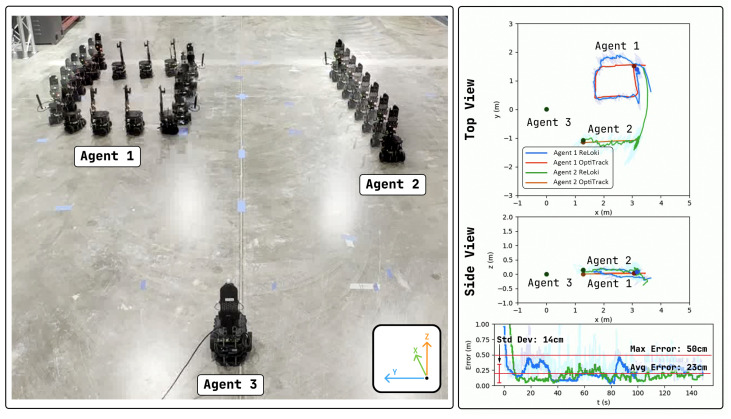
Localization experiment with RTA antenna on ReLoki. On the left, we have the composite of overlayed frames from the video captured during the experiment. Agent 1 is executing a rectangular motion and Agent 2 is executing a straight-line motion. On the right, we have the output from ReLoki as seen by Agent 3 as well as the Opti-Track data captured. We show both the raw estimation data, in a lighter color, and filtered data using a low-pass filter in a darker color.

In this experiment, we set up a three-robot system: Two agents (Agent 1 and 2) are in motion, while the third (Agent 3) is static. Agent 1 is executing a rectangular loop motion and Agent 2 is executing a straight-line trajectory, as shown in Figure 11. We record the relative position of the two agents in motion as measured by Agent 3. Note that the estimating agent can also be moving, but we leave it fixed to obtain a better visualization of the system in action.

We also show the filtered (using a low-pass filter) localization data output of the experiment along with the ground truth data captured from an overhead OptiTrack system in Figure 11. Here, we see very good congruence of the estimated trajectory to the ground truth, with a maximum localization error of 50cm, and an average localization error of 23cm. This localization error is slightly higher than the expected localization error of range-only trilateration-based UWB systems [30], but here we only use one localization pair which makes this system very decentralized. The localization performance is on par with the AoA-based 3D localization shown in [24], along with the added benefit of full 3D localization when using the RTA array. The localization errors can be further reduced by investigating the effects of a filter tuned to the specific motion type of the mobile platform, which will be one of the areas of investigation going forward.

For a system with two agents transmitting, with the CSMA scheme active, the maximum achievable sampling rate for each agent is 10 samples per second. In the experimental results showcased above, the localization happened as often as possible. To study the effects of scaling the system to a greater number of agents, we can artificially throttle the sampling rate. Table 1 shows the localization statistics with varying sampling rates. Here, we can clearly see the increase in localization error with a decreasing sampling rate. Thus, depending on the localization performance required for the application, the ReLoki system will be limited in the number of simultaneously transmitting agents.

#### 4.3.2. USA Antenna

**Figure 12:**
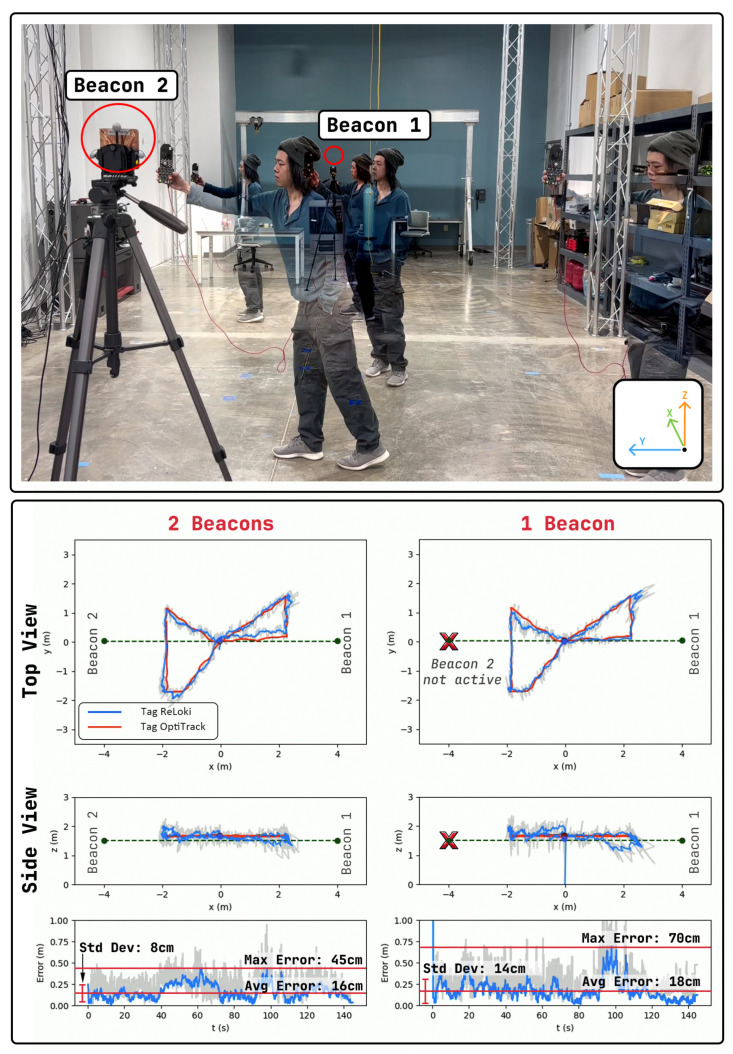
ReLoki beacon test. On the top, we show the experimental setup. Two beacons are placed 8m apart and the human operator moves the tag in a hour glass pattern. On the bottom, we show plots of the localization data along with the captured MoCAP data. Here, we show localization data with only one beacon active on the right side and both beacons active on the left. The unused beacon is marked with an “X”. We show the localization errors in both cases.

In this experiment, we use one tag hand-carried by a human operator and two beacons. We set up the ReLoki with the human operator as the transmitter and the ones on the beacons as the receivers. The human operator moves the tag in an hourglass pattern and transmits localization packets every 100ms to both beacons. All received data along with the sensed relative localization data from the beacons are piped onto a ground station. The received localization data are transformed to the coordinate frame of the first beacon and combined into a single estimate of the tag relative to this beacon. Finally, the localization data are filtered using independent low-pass filters on each beacon. The results of localization using only one beacon and a combination of two beacons are shown in Figure 12.

Looking at the results, we see that with only one beacon, the maximum localization is 70cm. The highest localization error in this case occurs when the tag is closer to the beacon and at a higher azimuth angle. Introducing the localization data from the second beacon reduces the localization errors to less than 45cm, with mean localization errors of 16cm. This performance is close to the range of trilateration-based localization with only three beacons [30], with the added benefit of using a smaller number of beacons.

**Remark** **2.** ***Multi-path effects:*** *The PDoA estimation method used by DW1000 modules is resilient to multi-path effects as long as the LoS path is not obstructed. The UWB modules have the capability of path separation when reflections are present, as shown in [5]. With the specific estimation running on the DW1000 modules that allow detection of the first path, when the LoS path is unobstructed, the phase difference measured is guaranteed to be the phase difference of the LoS signal. However, there will be a significant increase in the error when the LoS path is attenuated. In this case, the phase difference detected will be that of the first arriving reflected signal and will contribute to increased errors depending on the specific environment.*

## 5. Conclusions

In this paper, we propose a novel UWB-based relative localization system called ReLoki that leverages angle of arrival information from a four-antenna array in tandem with traditional ranging measurements to estimate the 3D relative position to any other participating ReLoki module. The paper discusses the system design and implementation for RTA, ROA, and USA antennas and shows that the RTA and ROA antenna arrays are better suited for full 3D relative localization systems and the USA array excels as an ad hoc mobile beacons. Both the RTA and ROA arrays have higher localization errors at higher elevation angles, but at the lower elevation angles between $-60^{\circ }$ and $60^{\circ }$, we achieve a localization error of less than 50cm. To combat these higher elevation errors, we need more research into antenna characteristics (e.g., effects of omnidirectionality and polarization) and other algorithmic improvements (e.g., per-antenna non-linear mapping or better antenna filtering). We also show the performance of the USA antenna array being better than the RTA and ROA arrays by leveraging the redundant antenna pairs to obtain better bearing estimates. We show a slightly better localization error with a maximum error of 45cm which is on par with the errors expected with trilateration, yet with a smaller number of beacons.

One of the main applications of the ReLoki platform is relative localization in indoor aerial systems like Lighter-than-Air (LTA) agents, where the low weight of the platform is an advantage. One of the main detriments here is the NLoS performance of the bearing estimations in the case of multi-agent systems and the characterization in a given environment. Our future focus will be on further studies on NLoS performance and non-linear estimation algorithms for better localization performance.

## Figures and Tables

**Table 1:** Effect of sampling rate on localization performance for RTA antenna. Here, we show the maximum localization error, average localization error, and the standard deviation for localization estimations of both robots in the experiment. Additionally, we also show the maximum number of agents supported with the specified sampling rate.

Sampling Rate	N. Agents	Max. Error (cm)	Avg. Error (cm)	Std. Dev. (cm)
10	3	50	23	14
5	5	48	26	21
2	10	52	31	29
1	20	58	36	32

## Data Availability

No Datasets were created in the process of this study.
